# Incidence of deep venous thrombosis (DVT) of the lower extremity in patients undergoing surgeries for ankle fractures

**DOI:** 10.1186/s13018-020-01809-0

**Published:** 2020-07-31

**Authors:** Luo Zixuan, Wei Chen, Yansen Li, Xiaomeng Wang, Weili Zhang, Yanbin Zhu, Fengqi Zhang

**Affiliations:** 1 Department of Foot and Ankle Surgery, The 3rd Hospital of Hebei Medical University, NO.139 Ziqiang Road, Shijiazhuang, 050051 Hebei People’s Republic of China; 2 Department of Orthopaedic Surgery, The 3rd Hospital of Hebei Medical University, NO.139 Ziqiang Road, Shijiazhuang, 050051 Hebei People’s Republic of China; 3 Key Laboratory of Biomechanics of Hebei Province, Shijiazhuang, 050051 Hebei People’s Republic of China

**Keywords:** Deep venous thrombosis, Ankle fractures, Epidemiology, Risk factors

## Abstract

**Objective:**

To investigate the incidence of postoperative deep venous thrombosis (DVP) in patients undergoing surgeries for ankle fractures and identify the associated risk factors.

**Methods:**

This was a retrospective study. A total of 1451 patients undergoing surgery of ankle fractures from January 2016 to June 2019 were included. The inpatient medical record system was inquired for data collection, including demographics, comorbidities, injury, and surgery-related data, and laboratory biomarkers. DVT of the lower extremity was diagnosed by routine Doppler examination. Univariate analyses and multivariate logistic regression analyses were used to identify the independent risk factors.

**Results:**

Among the 1451 patients, DVT was confirmed in 38 cases, indicating an incidence of 2.6%. DVT involved both the operated and non-operated limbs in 8 patients (21.1%). DVT involved superficial femoral vein in 4 cases (6.6%), deep femoral vein in 2 (3.3%), popliteal vein in 5 (8.2%), posterior tibial vein in 11 (18.0%), and peroneal vein in 39 (63.9%). The median interval between operation and diagnosis of DVT was 7 days. Six risk factors were identified to be independently associated with DVT, including age (10-year increase) (OR, 1.44), preoperative stay (delay of each day) (OR, 1.11), anesthesia (general vs regional) (OR, 3.51), lower hemoglobin level (OR, 2.02), total cholesterol > 5.2 mmol/L (OR, 3.20), and reduced lymphocyte count (OR, 3.16).

**Conclusion:**

These identified factors, although not easily modifiable, do help counsel patients about the risk of DVT and help individualized assessment of the risk factors and accordingly the risk stratification.

## Introduction

Ankle fracture is a common fracture type, with a population-based incidence of 37.1/100,000 person-years [1], representing 7.6% of all adult fractures [2]. Because of its special anatomical features, most of the ankle fractures involve the articular surface to varying degrees. The relatively thin skin and less soft tissue coverage more likely result in the swelling after fracture, and the post-trauma blood is often at high coagulation state. Moreover, surgical interventions (tissue dissection and stripping) and postoperative long-term immobilization of ankle joint also place regional blood at higher risk of formation of thrombosis. These constitute the theoretical pathological basis for thrombosis formation.

It is well established that deep venous thrombosis (DVT) of lower extremity following major orthopedic surgeries increased the risk of pulmonary emboli and mortality [3, 4]. By far, studies on DVT mainly focus on hip and knee joint arthroplasties [5, 6], the major orthopedics trauma (hip fracture, spinal injury, and others) [7–9], a specific population (elderly, patients with immobilization) [8], or prophylactic drug use (low-molecular-weight heparin, aspirin) [5, 6, 10, 11]. Specified at ankle fracture, however, there is still lack of epidemiologic data. SooHoo et al. [12] attempted to evaluate the risk factors associated with DVT following surgical treatment of ankle fractures, but the fewer potential factors included in the multivariate model might compromise the results. Shibuya et al. [13] evaluated the incidence of DVT and pulmonary embolism in foot and ankle trauma using the US national trauma data bank, but they did not separate the ankle fractures out for analysis. Basques et al. [14] used the ACS-NSQIP database and identified obesity, heart disease, and dependent functional status as independent factors associated with thromboembolic events after ankle fracture surgeries. Despite a national database used in these studies, the variability in data collection among the hospitals would be an issue.

Applicability of these results on DVT in other orthopedic surgeries or studies with small sample size might be poor, and the use of these results might result in a misdirected guidance in constructing a risk prediction model for DVT. Given these, we conducted this retrospective study, using data from inpatient records and laboratory tests, to evaluate the incidence rate of postoperative DVT and identify some risk factors associated with DVT.

## Methods

The study was reported in accordance with the STROCSS (Strengthening the Reporting of Cohort Studies in Surgery) guidelines. It was designed as a retrospective study and approved by the ethics committee of the 3rd Hospital of Hebei Medical University.

### Inclusion and exclusion criteria

Between January 2016 and June 2019, patients who underwent surgical treatment for acute ankle fractures in our hospital were included. The baseline characteristics and complications during hospitalization were obtained from the patient’s medical records. Inclusion criteria were patients aged 18 years or older, definite diagnosis of ankle fracture, surgical treatment (osteosynthesis), and complete data available in medical records. Exclusion criteria were pathological (metastatic) or old fracture (> 3 weeks since occurrence), open fracture, concurrent fractures in other locations, conservative treatment, incomplete medical records, current use of heparin, low molecular weight heparin (LMWH) or other anticoagulants due to chronic comorbidities, and preoperative diagnosis of DVT.

After admission, all patients received basic thromboprophylaxis, consisting of chemical (low molecular weight heparin (LMWH), 2500–4100 IU once daily, subcutaneous injection) and elevation of the injured lower extremity for each patient.

### Diagnosis of DVT

DVT was diagnosed in accordance with the Guideline for the Diagnosis and Treatment of Deep Vein Thrombosis (3rd edition) proposed by the Chinese Medical Association [15]. Ultrasound report was used to obtain the diagnostic information of DVT. During the postoperative hospitalization stay, in patients with lower limb swelling, pain, and tenderness in the rear and/or inner thighs, DVT was confirmed by duplex ultrasound: deep venous lumen obstruction or filling defect. Routine scanning was performed for the femoral common vein, superficial and deep femoral vein, popliteal vein, posterior and anterior tibial vein, and peroneal vein of bilateral lower extremities. Superficial or intermuscular vein thrombosis (soleal or gastrocnemius vein thrombosis) was excluded, due to their relatively less clinical significance [16, 17].

### Data collection

Electronic medical records (EMR), picture archiving and communication system (PACS), and operation reports were inquired for relevant data. The demographic data included age, gender, residence (urban or rural), body mass index (BMI), cigarette smoking, and alcohol consumption. The co-morbidities included hypertension, diabetes, ischemic heart disease, previous history of any surgery, and history of allergies to any medications, all of which were self-reported by patients. Fracture- and surgery-related data included fracture location (uni-, bi-, or trimalleolar fracture), injury mechanism (low- or high-energy trauma), accompanied dislocation, concurrent fracture, preoperative interval (between fracture occurrence and operation), American Society of Anesthesiologists (ASA) classification, anesthesia type, type of fracture reduction (open or closed), prophylactic use of antibiotics, surgical duration, bone graft, fixation type, intraoperative blood loss, perioperative blood transfusion, and postoperative use of drainage. The BMI (kg/m) was divided using the criteria recommended by the Chinese working group on obesity: normal (18.5–23.9), underweight (< 18.5), overweight (24.0–27.9), and obesity (≥ 28.0). Low-energy injury was defined as an injury caused by a fall from a standing height, and others such as fall from a height and motor accidents were high-energy injury.

The biomarkers from preoperative laboratory tests included total protein (TP) level, albumin (ALB) level, fasting blood glucose (FBG) level, preoperative red blood cell (RBC) count, white blood cell (WBC) count, neutrophile (NEUT) count, lymphocyte (LYM) count, neutrophile/lymphocyte rate (NLR), hemoglobin (HGB) level, hematocrit (HCT), platelet (PLT), red blood cell distribution width (RDW), serum total cholesterol (TC) level, triglyceride (TG) level, low-density lipoprotein (LDL-C) level, high-density lipoprotein (HDL-C) level, very low-density lipoprotein (VLDL) level, and D-dimer level. If patients had multiple laboratory tests before when DVT was diagnosed, laboratory tests closest to the diagnostic time point were chosen for data analysis.

### Statistical analysis

Continuous variables were expressed by mean and standard deviation (SD) and were evaluated by Student *t* test or Mann-Whitney *U* test. The categorical data were expressed as number and percentage (%) and were evaluated by chi-square or Fisher’s exact test. Multivariate logistics regression model was used to identify the independent risk factors associated with occurrence of DVT, using the stepwise backward elimination method. Variables with *p* < 0.10 were retained in the final model, and the correlation strength is indicated by odds ratio (OR) and 95% confidence interval (95% CI). The statistical test level was set as *p* < 0.05. Hosmer-Lemeshow (H-L) test was used to evaluate the fitting degree of the final model, and *p* > 0.05 represented the acceptable result. SPSS23.0 was used to perform all the tests (IBM, Armonk, New York, USA).

## Results

A total of 1451 patients with 1474 ankle fracture treated surgically were included for data analysis, consisting of 825 males and 626 females, with an average of 43.7 years (Sd, 15.1; range, 18–95; median, 43.0). Unimalleolar fracture accounted for 45.8% (673/1474), followed by trimalleolar fractures (471, 32.0%) and bimalleolar fractures (330, 22.2%). There were 264 (18.1%) fractures accompanied by dislocation or subluxation. The total hospitalization stay was 16.3 days in average (Sd, 14.6; median, 13; range, 2 to 137). The average preoperative hospitalization stay was 5.4 days (Sd, 3.5; range, 0–18; median, 5).

Of the 1451 patients, 38 developed a postoperative DVT, indicating an incidence rate of 2.6%. There was no thrombosis found in anterior tibial or femoral common vein. At the other 5 veins, a total of 61 clots were found, representing an average of 1.61 (range, 1 to 5) for each patient. DVT involved both the operated and non-operated limbs in 8 patients (21.1%). DVT involved only the non-operated limb in 5 patients (13.2%). The detailed DVTs involving veins were as follows: 4 in the superficial femoral vein, 2 in the deep femoral vein, 5 in the popliteal vein, 11 in the posterior tibial vein, and 39 in the peroneal vein. The interval between operation and diagnosis of DVT ranged from 1 to 26 days, with median at 7 days.

Table 1 presented the univariate analyses results. It was different between DVT and non-DVT patients in terms of age both in continuous (*p* < 0.001) and categorical variable (*p* = 0.001), diagnosis of diabetes mellitus, anesthesia type, preoperative stay, ASA classification, intraoperative bleeding, TP (< 60 g/L), ALB (< 35 g/L), TC (> 5.2 mmol/L), LYM (< 1.1 × 10^9^/L), RBC count, HGB level, HCT, PDW, RDW, and D-dimer level. DVT was associated with a significantly increased hospitalization stay of 6.9 days (23.0 vs 16.1 days).

**Table 1:** Univariate analyses of risk factors associated with DVT following ankle fracture surgeries

Variables	Number (%) of DVT (*n* = 38)	Number (%) of non-DVT (*n* = 1413)	*p*
**Gender (male)**	19 (50.0)	806 (57.0)	0.387
**Age (years)**	55.6 ± 14.6	43.4 ± 15.0	< 0.001
18–44	11 (28.9)	764 (54.1)	0.001
45–64	18 (47.4)	515 (36.4)	
65 or older	9 (23.7)	134 (9.5)	
**Living place**			0.789
Rural	23 (60.5)	777 (55.1)	
Urban	15 (39.5)	635 (44.9)	
**BMI (kg/m**^**2**^**)**	24.5 ± 3.6	25.9 ± 4.1	0.181
18.5–23.9	11 (28.9)	414 (29.3)	
< 18.5	2 (5.3)	25 (1.8)	
24.0–27.9	18 (47.4)	608 (43.0)	
≥ 28.0	7 (18.4)	366 (25.9)	
**Cigarette smoking**	9 (23.7)	212 (15.0)	0.142
**Alcohol consumption**	11 (28.9)	387 (27.4)	0.832
**Diabetes mellitus**	12 (31.6)	179 (12.7)	0.001
**Hypertension**	9 (23.7)	183 (13.0)	0.054
**Chronic heart disease**	3 (7.9)	65 (4.6)	0.343
**History of allergies to any medications**	8 (21.1)	202 (14.3)	0.243
**History of any surgery**	5 (13.2)	109 (7.7)	0.218
**Injury mechanism (high energy)**	10 (26.3)	284 (20.1)	0.347
**Fracture location**			0.757
Unimalleolar	17 (44.7)	656 (45.8)	
Bimalleolar	7 (18.4)	323 (22.5)	
Trimalleolar	14 (36.8)	454 (31.7)	
**Accompanied dislocation or subluxation**	9 (23.7)	258 (17.9)	0.361
**Preoperative stay**	7.7 ± 3.2	5.3 ± 3.6	< 0.001
**Total hospital stay**	23.0 ± 7.8	16.1 ± 14.7	0.004
**Anesthesia (general)**	19 (50.0)	271 (19.2)	< 0.001
**ASA class**			0.033
I	4 (10.5)	250 (17.7)	
II	25 (65.8)	1011 (71.5)	
III or above	9 (23.7)	152 (10.8)	
**Operation timing**			0.293
Day	38 (100)	1373 (97.2)	
Night	0	40 (2.8)	
**Type of fracture reduction**			0.780
Closed	1 (2.6)	49 (3.5)	
Open	37 (97.4)	1364 (96.5)	
**Fixation type**			
Screw or pins only	0	73 (5.2)	0.150
Plate and screws	38 (100)	1340	
**Bone grafting**	2 (5.3)	52 (3.7)	0.611
**Intraoperative bleeding**	254.2 ± 349.6	167.1 ± 213.9	0.014
**Perioperative blood transfusion (yes)**	3 (7.9)	44 (3.1)	0.101
**Surgical duration**	134.9 ± 74.4	130.7 ± 58.2	0.664
**TP (< 60 g/L)**	12 (31.6)	245 (17.3)	0.023
**ALB (< 35 g/L)**	10 (26.3)	171 (12.1)	0.009
**Intraoperative prophylactic use of antibiotics**	38 (100)	1332 (94.3)	0.129
**Postoperative use of antibiotics**	36 (94.7)	1304 (92.3)	0.575
**FBG (> 6.1 mmol/L)**	15 (39.5)	398 (28.2)	0.127
**TC (> 5.2 mmol/L)**	13 (34.2)	237 (16.8)	0.005
**TG (> 1.7 mmol/L)**	7 (18.4)	270 (19.1)	0.915
**LDL-C (> 3.37 mmol/L)**	11 (28.9)	250 (17.7)	0.075
**HDL-C (< 1.1 mmol/L)**	12 (31.6)	511 (36.2)	0.561
**VLDL (> 0.78 mmol/L)**	6 (15.8)	263 (18.6)	0.659
**WBC (> 10 × 10**^**9**^**/L)**	8 (21.1)	373 (26.4)	0.460
**NEUT (> 6.3 × 10**^**9**^**/L)**	17 (44.7)	603 (42.7)	0.726
**LYM (< 1.1 × 10**^**9**^**/L)**	20 (52.6)	273 (19.3)	< 0.001
**RBC (< lower limit)**	15 (39.5)	271 (19.2)	0.002
**HGB (< lower limit)**	15 (39.5)	269 (19.0)	0.002
**HCT (< lower limit)**	22 (57.9)	563 (39.8)	0.025
**PLT (> 300 × 10**^**9**^**/L)**	6 (15.8)	256 (18.1)	0.713
**PDW (> 18.1%)**	2 (5.3)	18 (1.3)	0.037
**RDW (> 16.5%)**	4 (10.5)	52 (3.7)	0.031
**D-dimer (> 0.3 mg/L)**	30 (81.1)	860 (60.9)	0.013

*DVT* deep venous thrombosis; *BMI* body mass index; *ASA* American Society of Anesthesiologists; *RBC* red blood cell, reference range: female, 3.5–5.0 × 10^12^/L, males, 4.0–5.5 × 10^12^/L; *HGB* hemoglobin, reference range: females, 110–150 g/L, males, 120–160 g/L; *FBG* fasting blood glucose; *HCT* hematocrit, 40–50%; *WBC* white blood cell, *NEUT* neutrophile; *LYM* lymphocyte; *PLT* platelet, 100–300 × 10^9^/L; *TP* total protein; *ALB* albumin; *RDW* red cell distribution width; *PDW* platelet distribution width; *TC* total cholesterol; *TG* triglyceride; *LDL-C*, low-density lipoprotein; *HDL-C* high-density lipoprotein; *VLDL* very low-density lipoprotein

In the multivariate model, the above-mentioned 17 variables together with hypertension (*p* = 0.054) and elevated LDL-C level (*p* = 0.075) were entered. Finally, six risk factors were identified to be associated with DVT, including age (10-year increase), preoperative stay (delay of each day), anesthesia (general vs regional), lower LGB level, TC > 5.2 mmol/L, and reduced LYM count (Table 2). As for other variables, we did not find the independent effect on DVT. The H-L test demonstrated the good fitness of the final model (*X*^2^ = 8.216, *p* = 0.413; Nagelkerke *R*^2^ = 0.220).

**Table 2:** Multivariate analyses of risk factors associated with DVT after ankle fracture surgeries

Variable	OR and 95% CI	*p* value
Age (increase of every 10 years)	1.44 (1.14 to 1.81)	0.002
Preoperative stay (delay of each day)	1.11 (1.02 to 1.21)	0.014
Anesthesia (general vs regional)	3.51 (1.73 to 7.13)	0.001
HGB < lower limit	2.02 (0.93 to 4.41)	0.078
TC (> 5.2 mmol/L)	3.20 (1.44 to 7.09)	0.004
Reduced LYM count (< 1.8 × 10^9^/L)	3.16 (1.55 to 6.41)	0.001

*DVT* deep venous thrombosis, *OR* odds ratio, *CI* confidence interval, *HGB* hemoglobin, *TC* total cholesterol, *LYM* lymphocyte

## Discussion

There remains scarce of epidemiologic data on DVT following surgery of ankle fractures. In this retrospective study, we evaluated the incidence rate of DVT during hospitalization period and identified some independent associated risk factors. Inclusion of a large sample of participants and multiple potential variables involving clinics, operation, and laboratory tests is the major advantage. This study may help counsel patients about the risk of DVT and help orthopedic surgeons in treatment decision-making, DVT risk evaluation and stratification, and taking targeted preventive measures.

Most of the previous studies focused on the preoperative DVT, and only a very few studies focused on the postoperative DVT, but reported the greatly varied results. Shibuya et al. [13] reported the incidence rate of acute DVT was 0.28% in 75,664 patients with foot and ankle trauma, using data from the National Trauma Data Bank. SooHoo et al. [12] used the California discharge database including 57,183 patients undergoing surgical treatment of ankle fractures and found the extremely low incidence rate of DVT that was 0.05%. In other studies with relatively small sample (100 to 2347), authors reported the comparable rates, 3.5% for DVT following surgical treatment [16] and 5.0% after plaster cast [18], which were consistent with ours (2.6%). These reported greatly varied figures for DVT in a specified ankle trauma population reflected the importance of study design, data resources, and also the differences in race and ethnics.

In this study, patients with DVT had a significant increased age of 12.2 years (55.6 vs 43.4) than those without DVT, and every increase of 10 years was associated with a 44% increased risk of DVT. Consistent with ours, the previous studies had demonstrated the similar dose-dependent effect of age on the occurrence of DVT following surgery of ankle trauma. Shibuya et al [13] found a converted 20% increased risk of DVT with every 10-year increment. Similarly, Stavem et al. [19] found the significant result when age was used in a continuous variable in the multivariate model. This can be explained by the vascular aging with age, such as damaged vascular endothelial cell and decreased fibrinolytic function and lower limb vein valve function [20]. However, it was not easy to confirm the cut-off value of age above which the risk of DVT was significantly increased, especially in the context of racial and ethnic differences. SooHoo et al. [12] found the age of 50–75 rather than > 75 years was associated with increased risk of DVT compared to those aged 50 years and younger. In another study, Duan et al. [20] used 40 years as the cut-off value and identified the independent effect of the advanced age on DVT occurrence. In this study, we divided the age into 3 groups based on the traditional criteria proposed by the WHO, but did not find the positive result in the multivariate model. It is possible that individual variation in vascular aging and sensitivity to thrombosis development should be a more worthy contributor than age itself.

The general anesthesia as an independent risk factor for DVT has been extensively reported in major surgeries, including hip fracture impairs and joint replacement [21, 22]. A meta-analysis of 21 randomized trials in total hip or knee [23] replacement demonstrated that general anesthesia was significantly associated with 2.48-fold increased risk of DVT, compared to regional anesthesia. However, specified at ankle fractures, general anesthesia as an independent factor for DVT was firstly reported in this study. This could be explained by the fact that, compared to general anesthesia, regional anesthesia allowed early mobility and improved the postoperative functional outcome and pain relief following surgical repair of ankle fractures [24]. In this study, the mean preoperative stay was 7.7 days in patients with DVT and 5.3 days in those without DVT (*p* < 0.001), and each extra day was associated with an 11% increased risk of DVT. However, the role of prolonged preoperative stay in development of DVT might be complex and should be treated dialectically. In fact, there were multiple factors that could affect the preoperative hospital stay, such as severity of trauma, number and severity extent of comorbidities, or the availability of operation room [25]. Also, the prolonged preoperative stay meant the longer-duration immobilization of the injured limb, which is the most common cause for DVT [26]. The relationship between preoperative stay and DVT occurrence requires further studies to investigate, especially in setting of adjustment for confounding factors as many.

The other 3 factors identified in this study were all from laboratory test: HGB level, TC level, and LYM count, and their relation to DVT had been discussed in the literature [27–31]. These biomarkers are a more likely indication of a low immune, systematic inflammation, and anemia status, but the stress effects from trauma cannot be ruled out [32]. In a study of humeral, femoral, and tibial diaphyseal fractures, authors observed significant changes of hematology panel biomarkers for any fracture location, compared to the controls [33]. In our study, if patients had multiple laboratory tests before when DVT was diagnosed, laboratory tests closest to the diagnostic time point were chosen for data analysis. Therefore, in consideration of the changes of biomarkers after trauma, our results could present more value in predicting DVT occurrence, compared to the commonly used admission biomarkers.

Several challenges and limitations in this study should be noted. Firstly, the retrospective design of this study compromised the accuracy in data collection, especially for comorbidities which were self-reported by patients. For patients with incomplete or non-available data in medical records, we had to exclude them, and in theory, it was randomized. Secondly, we could not include some factors that potentially affect the occurrence of DVT, such as immolization duration of the injured limb, prophylactic use of antithrombotic agents [16], or the use of a tourniquet during the operative procedure [34]. Thirdly, all DVTs were diagnosed during hospitalization, because DVT was not our routine follow-up content. Therefore, the incidence of DVT in this study was underestimated.

In summary, among the hospitalized patients undergoing surgery of ankle fractures, the incidence rate of postoperative DVT was 2.6%. Patients with DVT had a prolonged 1-week hospitalization stay than those without DVT. Age, prolonged preoperative stay, general anesthesia (vs regional), lower HGB level, hyperlipidemia, and reduced LYM count were identified as independent risk factors associated with DVT. Although most of them are not easily modifiable, they do help counsel patients about the risk of DVT and help individualized assessment of the risk factors and accordingly the risk stratification.

## Data Availability

All the data will be available upon motivated request to the corresponding author of the present paper.

## Abbreviations

DVT: Deep venous thrombosis
BMI: Body mass index
EMR: Electronic medical records
ASA: American Society of Anesthesiologists
SD: Standard deviation
OR: Odds ratio
RBC: Red blood cell
HGB: Hemoglobin
ALB: Albumin
FBG: Fasting blood glucose
HCT: Hematocrit
WBC: White blood cell
NEUT: Neutrophile
LYM: Lymphocyte
PLT: Platelet
TP: Total protein
PDW: Platelet distribution width
RDW: Red cell distribution width
TC: Total cholesterol
TG: Triglyceride
LDL-C: Low-density lipoprotein cholesterol
HDL-C: High-density lipoprotein cholesterol
VLDL: Very low-density lipoprotein cholesterol
